# Luminescence Efficiency Enhanced by Simple Substitutions on Donor and Acceptor in Radicals with Donor–Acceptor Structure

**DOI:** 10.3390/molecules30061191

**Published:** 2025-03-07

**Authors:** Shuang Gao, Jiahao Guan, Lintao Zhang, Xin Ai

**Affiliations:** 1 School of Materials Science and Engineering, Hainan University, No 58, Renmin Avenue, Haikou 570228, China; 2 Collaborative Innovation Center of Marine Science and Technology, Hainan University, No 58, Renmin Avenue, Haikou 570228, China; 3 Collaborative Innovation Center of Information Technology, Hainan University, No 58, Renmin Avenue, Haikou 570228, China

**Keywords:** organic luminescent radical, photoluminescence quantum efficiency, donor, acceptor, simple substitutions

## Abstract

Simple substitutions on the donor or acceptor units in radicals is an effective method to improve luminescent properties. However, the luminescence efficiency of radicals has not yet reached satisfactory levels through simple molecular structure modification. In this study, two [4-(N-Carbazolyl)-2,6-dichlorophenyl] bis(2,4,6-trichlorophenyl)methyl (Cz-TTM) radical derivatives (Mes_2_Cz-TTM, Mes_2_Cz-Mes_2_TTM) were synthesized and characterized by modifying the carbazole (donor) and tris-2,4,6-trichlorophenylmethyl radical (acceptor) units with 2,4,6-trimethylphenyl groups. The different substitutions showed varying influences on photoluminescence quantum efficiency (PLQE) compared to the Cz-TTM parent radical. The donor-only substitution suppressed the PLQE (39%) in Mes_2_Cz-TTM. In contrast, Mes_2_Cz-Mes_2_TTM exhibited a significantly higher PLQE of 92.6%, compared to the 68% PLQE of the Cz-TTM parent radical in toluene. Additionally, thermostability and photostability were improved with both donor and acceptor substitutions. The photophysical properties, molecular orbitals, and electrochemical behaviors were also systematically explored. This strategy provides a feasible approach to achieve high luminescence efficiency in radicals through simple substitutions on donor and acceptor units.

## 1. Introduction

**Figure 1:**
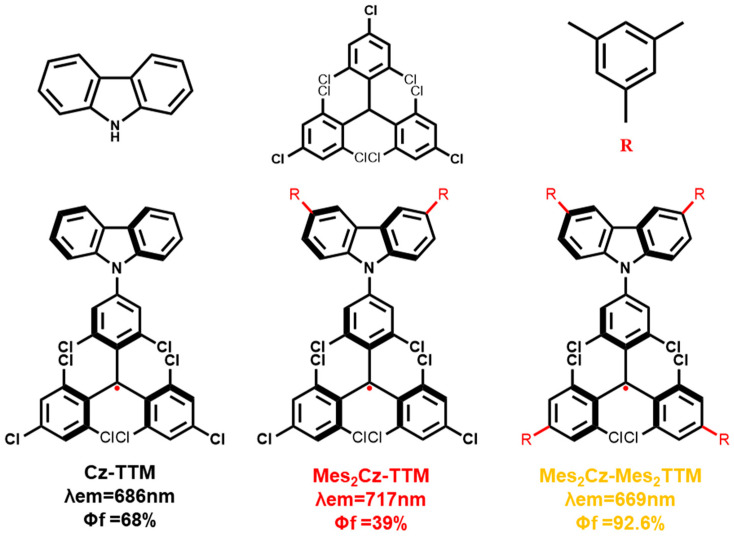
Molecular structures of Cz-TTM, Mes_2_Cz-TTM, and Mes_2_Cz-Mes_2_TTM.

Due to the distinctive doublet emission mechanism, organic radicals possess inherent advantages as luminescent materials. With the exception of a few newly developed luminescent radicals, the chlorinated triphenylmethyl (trityl) radicals and their derivatives have been extensively studied [1,2,3,4,5,6,7]. These include tris(2,4,6-trichlorophenyl)methyl (TTM) radicals [8,9,10,11,12,13,14,15,16,17,18,19,20,21], perchloro triphenylmethyl (PTM) radicals [22,23,24], pyridyl-containing triphenylmethyl (PyBTM) radicals [25,26,27,28,29,30,31], (N-carbazolyl) bis(2,4,6-trichlorophenyl)methyl (CzBTM) radicals [32,33,34], and so on. Traditional PTM or TTM derivatives typically feature donor–acceptor structures, with an electron-rich donor (carbazole or triphenylamine, etc.) and an electron-deficient acceptor (PTM or TTM radical core). Some studies had documented significant enhancements in the luminescent properties of these radicals [35,36,37,38,39,40,41,42,43]. However, radicals with high photoluminescence quantum efficiency (PLQE) are still relatively rare compared to other types of organic luminescent materials. Luis Juliá et al. first incorporated carbazole (electron donor) into a TTM radical discovering an efficient red light-emitting radical [17]. However, the terminal modifications of the carbazole donor unit with phenyl derivatives or halogen atoms had a minimal impact on the luminescent efficiency of the radicals in latter works [42,44,45]. In contrast, direct bonding to the radical acceptor core was proved to be more effective [35,36,39,40,41]. However, due to the low luminescence efficiency of the parent radicals (PyBTM, CzBTM), the improvement in PLQE did not meet expectations. Recently, Li et al. reported nearly 100% PLQE radical emitters by fine-tuning the effective donor-acceptor distance in [4-(N-Carbazolyl)-2,6-dichlorophenyl] bis(2,4,6-trichlorophenyl)methyl (Cz-TTM) radical analogues [46]. This suggests that the design strategy of modifying the donor-acceptor radical system to achieve high PLQE is promising. In this study, Cz-TTM with 68% PLQE in toluene was used as the parent radical. Two new Cz-TTM derivatives (Mes_2_Cz-TTM, Mes_2_Cz-Mes_2_TTM) were synthesized by modifying the carbazole donor and TTM radical acceptor unit with 2,4,6-trimethylphenyl (Mes) (Figure 1). In Mes_2_Cz-TTM, the substitution of Mes on the carbazole donor significantly lowered the luminescence efficiency (39%) compared to the minimal impact with benzene substitution (electron-donating) [44] and the negative impact with the halogen atom (electron-withdrawing) [42,45]. Surprisingly, Mes_2_Cz-Mes_2_TTM, with further substitution on the TTM radical acceptor, achieved an excellent PLQE (92.6%). Additionally, both radicals exhibited enhanced photostability and thermostability due to the introduction of donor and acceptor substitutions. This simple molecular design strategy is proved to be highly effective in achieving luminescent radicals with excellent luminescence efficiency.

## 2. Results and Discussion

### 2.1. Synthesis and Structure Characterization

Mes_2_Cz-TTM and Mes_2_Cz-Mes_2_TTM were synthesized using commercially available reagents. The detailed synthetic routes were described in the Supporting Information (Figure S1). The molecular structures and compositions of these target radicals were characterized by MALDI-TOF (Figure S2) and Fourier transform infrared spectra (FT-IR) (Figure S3). The absorption peaks ranging from 1600 cm^−1^ to 1400 cm^−1^ mainly originated from benzene rings and the bonds that connect the central carbon atom of the radical to the adjacent benzene rings. The presence of the unpaired electron in the radicals (g values around 2.002–2.003) was confirmed by electron paramagnetic resonance (EPR) (Figure S4).

### 2.2. Photophysical Properties

**Figure 2:**
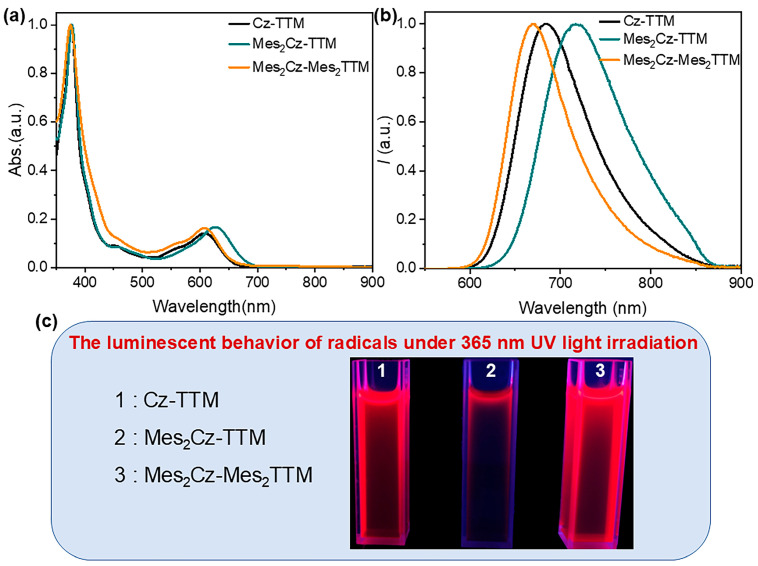
(**a**) The normalized UV-Vis absorption spectra of Cz-TTM, Mes_2_Cz-TTM, and Mes_2_Cz-Mes_2_TTM in toluene solution (1 × 10^−^^5^ M) at room temperature; (**b**) the normalized PL spectra of Cz-TTM, Mes_2_Cz-TTM, and Mes_2_Cz-Mes_2_TTM in toluene solution (1 × 10^−^^5^ M) at room temperature; and (**c**) the luminescent behavior of radicals under 365 nm UV light irradiation in toluene solvent.

The ultraviolet-visible (UV-Vis) absorption spectra and photoluminescence (PL) spectra of Mes_2_Cz-TTM and Mes_2_Cz-Mes_2_TTM were measured in toluene solvent (1 × 10^−5^ M) (Figure 2a,b). The Cz-TTM parent radical was also measured as a reference. Similar to Cz-TTM, the absorption band around 376 nm was attributed to the characteristic absorption of carbon-centered radicals. A broad and weaker absorption band at longer wavelengths (608 nm) was primarily due to the intramolecular charge transfer (CT) state. As Mes was introduced, increasing the electron-donating ability of the carbazole unit, the enhanced charge transfer effect resulted in a red shift of the absorption band (626 nm) from the carbazole donor to the TTM radical acceptor in Mes_2_Cz-TTM. When Mes was attached to the dichlorophenyl group of the TTM radical core, the long-wavelength absorption band shifted back to the initial wavelength (610 nm), which was associated with the weakened donor-acceptor character due to the decreased electron-accepting ability of the TTM radical core in Mes_2_Cz-Mes_2_TTM. On the other hand, the PL spectra of the target radicals followed a similar trend to the UV-Vis absorption spectra. Both radicals exhibited red fluorescence emission (Figure 2c). The emission band of Mes_2_Cz-TTM exhibited a bathochromic shift (717 nm) due to the effect of donor substitution. With the substitution of the TTM radical acceptor, a blue shift in emission (669 nm) was observed, resulting from the weaker electron-accepting TTM radical core. Moreover, we concluded that the effect of acceptor substitution on the shift in emission was more pronounced. The absorption bands showed no solvent dependence behavior, while the emission bands shifted to longer wavelengths with increasing solvent polarity, indicating the CT excited-state character (Figures S5 and S6).

**Figure 3:**
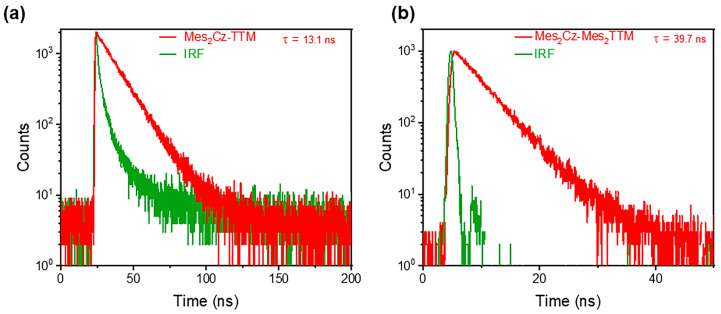
Transient fluorescence decay of Mes_2_Cz-TTM (**a**) and Mes_2_Cz-Mes_2_TTM (**b**) in toluene solution (1 × 10^−^^5^ M).

The absolute PLQE values in toluene were measured using an integrating sphere. The different substitutions on the donor and acceptor units resulted in opposite trends. The Mes substitution significantly suppressed the PLQE in Mes_2_Cz-TTM (39%). The suppression on luminescence efficiency was more obvious than the previous Cz-TTM derivatives, especially the terminal modification of carbazole with phenyl derivatives. The simple substitution on carbazole donor may not be an effective method. However, when the additional Mes group was directly attached to the TTM radical core, the PLQE of Mes_2_Cz-Mes_2_TTM (92.6%) dramatically increased compared to the PLQE of Cz-TTM (68%). To explain these results, the transient photoluminescence decay spectra were measured in toluene. Mes_2_Cz-TTM radical exhibited shorter lifetime and Mes_2_Cz-Mes_2_TTM radical exhibited a longer lifetime compared to Cz-TTM (Figure 3). The radiative rate constant (k_r_) and non-radiative rate constant (k_nr_) for Mes_2_Cz-TTM and Mes_2_Cz-Mes_2_TTM were also calculated according to Equations (1) and (2) (Table 1). The k_r_ of Cz-TTM, Mes_2_Cz-TTM, and Mes_2_Cz-Mes_2_TTM were on the same order, which was due to the similar transition oscillator strength (Table S4). Therefore, the k_nr_ played the main role in determining luminescence efficiency. Interestingly, distorted molecular space structure did not lead to lower k_nr_ in Mes_2_Cz-TTM. In contrast, the possible flipping motions may cause larger thermal deactivation paths. Mes_2_Cz-Mes_2_TTM showed lower k_nr_ than Cz-TTM. On the one hand, the direct attachment of Mes to TTM radical acceptor impeded the vibronic coupling of the luminescence core. On the other hand, the larger energy gap suppressing the internal conversion to the ground state also resulted in lower k_nr_.

(1) $$\Phi =\frac{k_{r}}{k_{r}+k_{nr}}$$

(2) $$\tau =\frac{1}{k_{r}+k_{nr}}$$

### 2.3. Stability

The thermostabilities of the radicals were compared using thermogravimetric analysis (TGA) under ambient and nitrogen atmospheres, respectively (Figure S7). The differences in decomposition temperatures (T_d_, corresponding to 5% weight loss) were attributed to the different substitution sites. Mes_2_Cz-TTM with a modified carbazole unit exhibited poorer stability than Cz-TTM. The cause for the significant decrease in stability was unclear. In contrast, the higher decomposition temperature of Mes_2_Cz-Mes_2_TTM was due to the substituents replacing the reactive chlorine sites in the TTM radical core, which are key to its reactivity.

**Figure 4:**
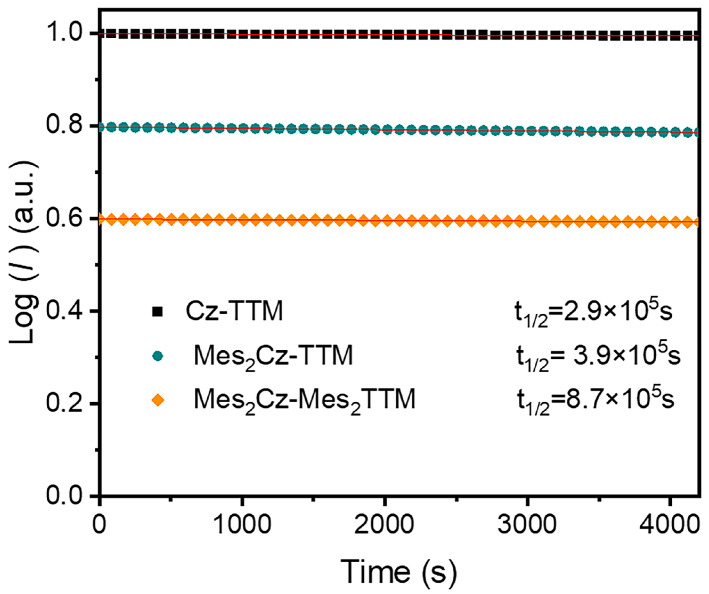
Photostability of Cz-TTM, Mes_2_Cz-TTM, and Mes_2_Cz-Mes_2_TTM.

Radicals were also monitored under continuous irradiation with a 375 nm xenon lamp in toluene to measure the half-life (t_1/2_) values (Figure 4). Compared to Cz-TTM (2.9 × 10^5^ s), a longer t_1/2_ for Mes_2_Cz-TTM (3.9 × 10^5^ s) and Mes_2_Cz-Mes_2_TTM (8.7 × 10^5^ s) indicated stronger photostabilities. As the number of substitution groups increased, photostability also showed an increasing trend (Cz-TTM < Mes_2_Cz-TTM < Mes_2_Cz-Mes_2_TTM). The enhanced photostability was attributed to the more stable excited state resulting from larger conjugation and steric hindrance. Also, the introduction of 2,4,6-trimethylphenyl can effectively reduce the reactions at active sites, such as the 3,6 positions of the carbazole and the ortho positions of the radical benzene ring.

### 2.4. Electrochemistry

**Figure 5:**
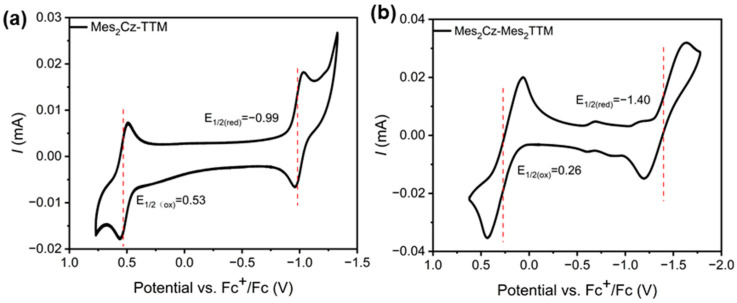
Cyclic voltammetry (CV) curves of Mes_2_Cz-TTM (**a**) and Mes_2_Cz-Mes_2_TTM (**b**) in dichloromethane. (The red lines indicate the positions of the half-wave potential.)

Cyclic voltammetry (CV) was performed to assess the redox potentials of Mes_2_Cz-TTM and Mes_2_Cz-Mes_2_TTM. Both compounds exhibited reversible redox behavior in dichloromethane (Figure 5). In Mes_2_Cz-TTM, the standard reduction potential and oxidation potential (−0.99 V, 0.53 V) were lower than Cz-TTM (−0.98 V, 0.55 V). The minimal decrease in potential was attributed to the weak electron-donating effect of the Mes fragments due to the torsion angles between the carbazole and Mes. The reduction/oxidation peaks also shifted to obviously lower potentials (−1.40 V/0.26 V) in Mes_2_Cz-Mes_2_TTM. The result was attributed to the direct connection between Mes and the TTM radical core, which decreases its electron-deficient character. The energy levels of the α-SOMO and β-SUMO were calculated based on the redox potentials. Based on results, the energy levels (α-SOMO, β-SUMO) of Mes_2_Cz-TTM (−5.33 V, −3.81 V) were higher than Cz-TTM (−5.35 V, −3.82 V). Mes_2_Cz-Mes_2_TTM (−5.06 V, −3.40 V) was significantly higher than Cz-TTM and Mes_2_Cz-TTM. The data suggested that the donor and acceptor substitutions increased the frontier energy levels of the radicals. The 20 cycles of CV scans demonstrated that both radicals had excellent electrochemical stability (Figure S8).

### 2.5. Theoretical Calculations

To investigate the effects of donor and acceptor substitutions on the frontier molecular orbitals (MOs) and electron density distribution of Cz-TTM type radicals, density functional theory (DFT) calculations (B3LYP/6-31G(d,p)) were performed. The optimized structures of Cz-TTM, Mes_2_Cz-TTM, and Mes_2_Cz-Mes_2_TTM revealed negligible change (Tables S2 and S3). The donor and acceptor substitutions did not significantly affect the structure of the Cz-TTM core. Although the dihedral angles between Mes and the aromatic nuclei of carbazole or the TTM radical core remained distorted, the donor and acceptor substitutions also resulted in a modest shift in the absorption and emission bands.

**Figure 6:**
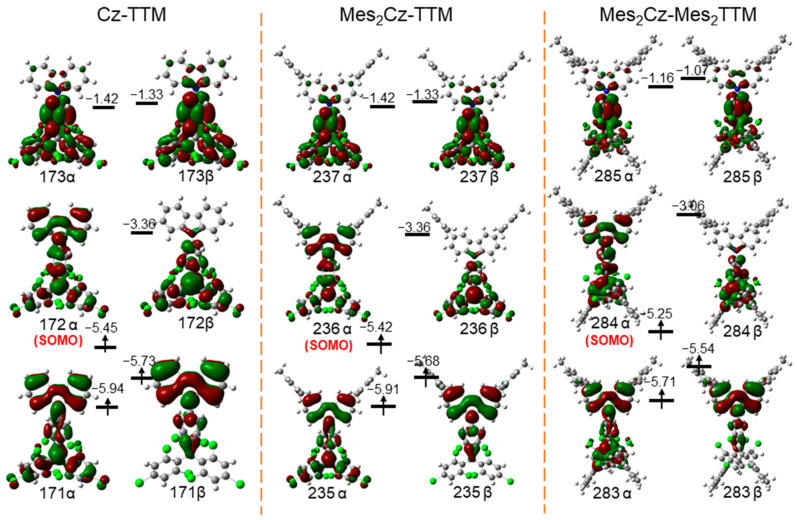
Frontier molecular orbitals calculated by DFT calculations of Cz-TTM, Mes_2_Cz-TTM, and Mes_2_Cz-Mes_2_TTM. (Red and dark green distributions on molecule represent electron cloud distributions; green, blue, gray, and white balls represent Cl, N, C, and H atoms, respectively.)

Mes_2_Cz-TTM and Mes_2_Cz-Mes_2_TTM exhibited similar spin-density distributions (Figure S9). The unpaired electron, primarily located on the TTM radical core and partially extending to the carbazole unit, was not obviously affected by the donor and acceptor substitutions. The MOs of Cz-TTM, Mes_2_Cz-TTM, and Mes_2_Cz-Mes_2_TTM showed that the positions of the singly occupied molecular orbital (α-SOMO) and singly unoccupied molecular orbital (β-SUMO) remained largely unchanged. The α-SOMO was mainly located on the TTM radical core and the carbazole unit, while the β-SUMO was distributed over the TTM radical core, with slight extension to the carbazole unit (Figure 6). The energy levels of the α-SOMO and β-SUMO in Mes_2_Cz-TTM (−5.42 eV, −3.36 eV) were slightly higher than Cz-TTM (−5.45 eV, −3.36 eV) with the substitution on the carbazole unit. Mes_2_Cz-Mes_2_TTM exhibited significantly higher α-SOMO (−5.25 eV) and β-SUMO (−3.06 eV) energy levels due to the additional substitution on the TTM radical acceptor. The results were consistent with the electrochemistry. Time-dependent DFT (TD-DFT) calculations (B3LYP/6-31G(d,p)) were also performed to further explore the excited states. The results showed that the doublet excited states (D_1_) of Cz-TTM, Mes_2_Cz-TTM, and Mes_2_Cz-Mes_2_TTM originated from the 171β→172β, 235β→236β, and 283β→284β transitions, respectively (Table S4). The increasing trend in energy levels (Cz-TTM < Mes_2_Cz-TTM < Mes_2_Cz-Mes_2_TTM) and transition energies of D_1_ (Mes_2_Cz-TTM < Cz-TTM < Mes_2_Cz-Mes_2_TTM) confirmed the shift trend in the emission bands.

## 3. Materials and Methods

Mes_2_Cz-TTM and Mes_2_Cz-Mes_2_TTM radicals were prepared according to the detailed synthesis routes in Supporting Information. All raw materials and chemical reagents used in this work were procured from ERNEGI and Xilong Science Co., Ltd. (Shanghai, China) without further purification. The mass spectra measurement was performed on MALDI-TOF (ion source 1: 20 kV, ion source 2: 17.65 kV, lens: 8 kV, reflector: 20.98 kV, reflector 2: 11.09 kV, pulsed ion extraction: 170 ns, DCTB as the matrix in the MALDI-TOF testing). Infrared (IR) spectra were collected on a BRUKER TENSOR 27 spectrophotometer (Bruker Corporation, Bruck, Germany) using KBr pellets. The EPR testing was finished with Bruker A320 spectrometer (Bruker Corporation, Bruck, Germany) (radicals in solid state and 0.1 M dichloromethane solution, sweep width: 100 G, frequency: 9.852902 GHz, power: 19.83 mW, resolution in X: 1024, number of X-scans: 1, temperature: room temperature). The UV-Vis spectra were obtained with a Shimadzu UV-1900i UV-Vis spectrometer (Shimadzu (Suzhou) Instruments Co., Ltd., Suzhou, China) (slit width: 2 um). The photoluminescence spectra were acquired with the Shimadzu RF-6000 spectrometer (Shimadzu Corporation, Kyoto, Japan) (excitation bandwidth: 3.0 nm; emission bandwidth: 3.0 nm; scan speed: 600 nm/min). The PL decays were measured on the Edinburgh FLS1000 spectrometer (Edinburgh Company, Edinburgh, UK), and the absolute PLQEs were measured on the identical apparatus via the integrating sphere method (slit width: 50 um). The DFT and TD-DFT calculations were run on Gaussian16 C.02 commercial software [47]. The TGA tests in air and nitrogen atmospheres were performed with TA INSTRUMENTS Q600 instrument (PERKINELMER, Waltham, MA, USA) (heating rate: 10 °C/min). The electrochemistry data were obtained with CH Instruments CHI660E electrochemical analyzer (scan rate: 300 mVs−1; supporting electrolyte: 0.1 M (Bu4N)PF6; working, counter, and reference electrodes: glassy graphite, platinum, and Ag/AgCl; ferrocene was added as an internal standard in the measurement). The photostability was tested under continuous xenon lamp irradiation, using the Shimadzu RF-6000 spectrometer (Shimadzu Corporation, Kyoto, Japan) (excitation bandwidth: 3.0 nm, emission bandwidth: 3.0 nm, integration time: 10 ms).

### 3.1. Synthesis of Compound Mes_2_Cz-TTM

Mes_2_Cz-TTM: MALDI-TOF (*m*/*z*): [M]+ calcd. for C_49_H_34_C_l8_N˙, 920.42; found, 920.75. IR(KRI): 2960(s), 2925(m), 2867(m), 1610(m), 1575(m), 1504(m), 1458(m), 1371(m), 1319(w), 1265(m), 1243(w), 1189(w), 1083(w), 1035(w), 877(m), 804(m).

### 3.2. Synthesis of Compound Mes_2_Cz-Mes_2_TTM

Mes_2_Cz-Mes_2_TTM: MALDI-TOF (*m*/*z*): [M]+ calcd. for C_67_H_56_C_l6_N˙, 1087.89; found, 1088.06. IR(KRI): 2952(m), 2920(m), 2856(w), 1612(m), 1577(m), 1506(s), 1473(m), 1375(m), 1284(w), 1263(w), 1220(w), 1188(w), 1033(w), 875(m), 852(m), 808(w), 742(w).

## 4. Conclusions

In summary, we successfully designed and synthesized two Cz-TTM radical derivatives (Mes_2_Cz-TTM and Mes_2_Cz-Mes_2_TTM) by incorporating Mes groups into the carbazole donor and TTM radical acceptor units of Cz-TTM. The two radicals exhibited distinct luminescence efficiencies due to the different substitution positions. The sole donor substitution on the carbazole unit led to a reduced PLQE in Mes_2_Cz-TTM (39%). However, Mes_2_Cz-Mes_2_TTM achieved a significantly higher PLQE of 92.6%, which is notably greater than that of Cz-TTM (68%). The introduction of substitutions on the donor and acceptor sites also improved the thermostability and photostability. Theoretical calculations were carried out to explain the differences in the luminescent properties. This work demonstrates that simple modifications on the donor and acceptor units in radicals with donor–acceptor structure are effective in enhancing the PLQE of luminescent radicals.

## Figures and Tables

**Table 1:** Photophysical parameters of Cz-TTM, Mes_2_Cz-TTM, and Mes_2_Cz-Mes_2_TTM in toluene.

Radicals	λ_a_ (nm) ^[a]^	λ_f_ (nm) ^[a]^	Φ_f_ (%) ^[b]^	τ (ns) ^[c]^	k_r_ (s^−1^)	k_nr_ (s^−1^)
Cz-TTM	376,608	686	68	24.9	27 × 10^6^	13.2 × 10^6^
Mes_2_Cz-TTM	377,626	717	39	13.1	29 × 10^6^	47 × 10^6^
Mes_2_Cz-Mes_2_TTM	375,610	669	92.6	39.7	34 × 10^6^	2.8 × 10^6^

^[a]^ UV-Vis spectra, ^[b]^ the integrating sphere method, and ^[c]^ transient fluorescence decay spectrum.

## Data Availability

The original contributions presented in the study are included in the article/Supplementary Material; further inquiries can be directed to the corresponding author.

## Supplementary Materials

The following supporting information can be downloaded at https://www.mdpi.com/article/10.3390/molecules30061191/s1, Figure S1: The synthetic routes of Mes_2_Cz-TTM and Mes_2_Cz-Mes_2_TTM; Figure S2: Mass spectrometry of Mes_2_Cz-TTM and Mes_2_Cz-Mes_2_TTM; Figure S3: FT-IR spectra of Mes_2_Cz-TTM and Mes_2_Cz-Mes_2_TTM; Figure S4: EPR spectra of Mes_2_Cz-TTM and Mes_2_Cz-Mes_2_TTM in dichloromethane solution and powder at room temperature. Figure S5: UV-Vis absorption spectra of Mes_2_Cz-TTM in solutions of different polarities (10^−5^ M); Figure S6: UV-Vis absorption spectra of Mes_2_Cz-Mes_2_TTM in solutions of different polarities (10^−5^ M); Figure S7: TGA curve of Mes_2_Cz-TTM and Mes_2_Cz-Mes_2_TTM; Figure S8: Cyclic voltammetry (CV) curves of Mes_2_Cz-TTM and Mes_2_Cz-Mes_2_TTM for multiple (20-turn) cycles.; Figure S9: Spin densities of Cz-TTM, Mes_2_Cz-TTM and Mes_2_Cz-Mes_2_TTM by DFT calculations.; Table S1: Redox potentials and corresponding orbital energy levels calculated theoretically and measured experimentally of Cz-TTM, Mes_2_Cz-TTM, and Mes_2_Cz-Mes_2_TTM; Table S2: The values of characteristic torsion angles in radical molecules in theoretical calculations; Table S3: The bond lengths of radical molecules in theoretical calculations; Table S4: The parameters corresponding to the D_1_ transition in the TD-DFT calculation results of radicals.
