# Supplementary material for: Luminescence Efficiency Enhanced by Simple Substitutions on Donor and Acceptor in Radicals with Donor–Acceptor Structure
Source: Molecules. 2025 Mar 7;30(6):1191. doi: 10.3390/molecules30061191 (PMC11944918; doi:10.3390/molecules30061191)
Supplement: Supplementary file 1 [file molecules-30-01191-s001.zip › molecules-3493086-supplementary.pdf]

molecules

Supporting information

Luminescence efficiency enhanced by simple substitutions on donor and acceptor in radicals with donor–acceptor structure

Shuang Gao [1,†], Jiahao Guan [1,†], Lintao Zhang [1,2,*] and Xin Ai [1,2,3,*]

[1] School of Materials Science and Engineering, Hainan University, No 58, Renmin Avenue, Haikou 570228, China
[2] Collaborative Innovation Center of Marine Science and Technology, Hainan University, No 58, Renmin Avenue, Haikou 570228, China
[3] Collaborative Innovation Center of Information Technology, Hainan University, No 58, Renmin Avenue, Haikou 570228, China
* Correspondence: zhanglintao@hainanu.edu.cn (L.Z.); aixin133@hainanu.edu.cn (X.A.)
† These authors contributed equally to this work.

Contents

Figure S1 The synthetic routes of Mes_2Cz-TTM and Mes_2Cz-Mes_2TTM……………………………………………….……...3

Figure S2 Mass spectrometry of Mes_2Cz-TTM and Mes_2Cz-Mes_2TTM……………………………………………….….……4

Figure S3 FT-IR spectra of Mes_2Cz-TTM and Mes_2Cz-Mes_2TTM……..………………………………………………….….……4

Figure S4 EPR of Mes_2Cz-TTM and Mes_2Cz-Mes_2TTM at room temperature…….….……….…………………………………...5

Figure S5 UV-Vis absorption spectra of Mes_2Cz-TTM in solutions of different polarities (10^{-5} M) ……….….………………5

Figure S6 UV-Vis absorption spectra of Mes_2Cz-Mes_2TTM in solutions of different polarities (10^{-5} M) …….….……..…6

Figure S7 TGA curve of Mes_2Cz-TTM and Mes_2Cz-Mes_2TTM……..……………………………………………….….………6

Figure S8 Cyclic Voltammetry (CV) curves of Mes_2Cz-TTM and Mes_2Cz-Mes_2TTM for multiple (20-turn) cycles……....6

Table S1: Redox potentials and corresponding orbital energy levels calculated theoretically and measured experimentally of Mes_2Cz-TTM and Mes_2Cz-Mes_2TTM……..………………………………………………….………………...7

Table S2: The values of characteristic torsion angles in radical molecules in theoretical calculations…….….…………....7

Table S3: The bond lengths of radical molecules in theoretical calculations……………………….………………………...8

Figure S9 Spin densities of Cz-TTM, Mes_2Cz-TTM and Mes_2Cz-Mes_2TTM by DFT calculations…….….………………….....8

Table S4: The parameters corresponding to the D_1 transition in the TD-DFT calculation results of radicals……………..8

Appendix (Calculation data) …..………………………………………….…………….….……………….…………………...9

Supporting Reference……………………………………………………………………………………………………..15

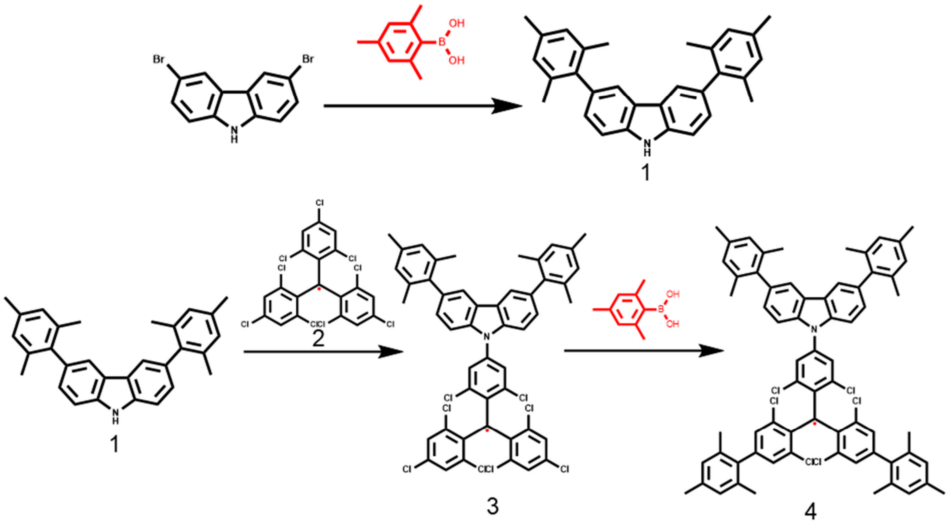

Figure S1. The synthetic routes of Mes_2Cz-TTM and Mes_2Cz-Mes_2TTM

Synthesis of compound 1

A mixture of 3,6-dibromocarbazole (3.25 g, 10 mmol), 2,4,6-trimethylphenylboronic acid (4.9 g, 30 mmol), palladium acetate (0.11 g, 0.5 mmol), tri(o-tolyl) phosphine (0.3 g, 1 mmol), and barium hydroxide monohydrate (5.1 g, 30 mmol) was added to a 250 mL two-necked flask. Under a nitrogen atmosphere, 81 mL of super-dry ethylene glycol dimethyl ether and 13 mL of ultrapure water were added. The reaction was carried out at 80°C for 8 hours. After completion, the reaction mixture was cooled to room temperature, and the crude product was filtered through Celite. The filtrate was extracted with dichloromethane (80 mL × 3), and the solvent was removed to yield a silica gel-like residue. The residue was purified by column chromatography using a petroleum ether: ethyl acetate (20:1) solvent system. The product was collected, and after evaporation of the solvent, 2.2 g of the product was obtained, yielding 45%. 1H NMR (400 MHz, Chloroform-d) δ 7.43 (s, 2H), 10.44 (s, 1H). MALDI-TOF (m/z): [M]+ calcd. for $C_{42}H_{33}C_{14}N_2$, 720.795; found, 720.783.

Synthesis of compound 2（TTM）

Tris(2,4,6-trichlorophenyl) methane radical (TTM) was synthesized following previous procedures.[1]

Synthesis of compound 3 (Mes_2Cz-TTM)

Under argon atmosphere, TTM（0.54 g，1 mmol）, Mes_2Cz（0.4 g，1 mmol）, $CsCO_3$（0.34 g，1 mmol） were mixed in anhydrous DMF (40 ml). The mixture was refluxed at 160℃ for 12 h. After cooled slowly to room temperature, the mixture was poured into HCl solution（100 ml，0.20 M）and filtrated. The sediment was extracted with dichloromethane by three times. The combined organic layer was washed with sodium bicarbonate solution and dried with anhydrous sodium sulfate. After solvent was removed under reduced pressure and the crude product was purified by flash column chromatography using petroleum ether/dichloromethane (silica gel, 10:1, v/v) as eluent. Red solid was obtained in 22 % yield (0.20 g, 0.21 mmol). MALDI - TOF (m/z): [M]+ calcd. for $C_{49}H_{34}C_{18}N^{\cdot}$, 920.42; found, 920.75. IR(KRI): 2960(s), 2925(m), 2867(m), 1610(m), 1575(m), 1504(m), 1458(m), 1371(m), 1319(w), 1265(m), 1243(W), 1189(w), 1083(w), 1035(w), 877(m), 804(m).

Synthesis of compound4 (Mes_2Cz-Mes_2TTM)

Under argon atmosphere, Mes_2Cz-TTM（0.45 g，0.49 mmol）, 2,4,6-Trimethylphenylboronic（0.24 g，1.47 mmol）, Pd(dtbpf)Cl_2（0.12 g，0.18 mmol）were mixed in 30 ml emulsion (K-EL 2wt%: toluene, 9:1 v/v). The mixture was heated to 70℃ and 6 ml triethylamine was added. The mixture was heated at 70℃ for 48 h. After cooled slowly to room temperature, the mixture was

extracted with dichloromethane by three times. The combined organic layer was washed with sodium bicarbonate solution and dried with anhydrous sodium sulfate. After solvent was removed under reduced pressure and the crude product was purified by flash column chromatography using petroleum ether/dichloromethane (silica gel, 10:1, v/v) as eluent. Red solid was obtained in 22 % yield (0.20 g, 0.18 mmol). MALDI‐TOF (m/z): [M]+ calcd. for $C_{67}H_{56}Cl_{6}N^{\cdot}$, 1087.89; found, 1088.06. IR(KRI): 2952(m), 2920(m), 2856(w), 1612(m), 1577(m), 1506(s), 1473(m), 1375 (m), 1284(w), 1263(w), 1220(W), 1188(W), 1033(w), 875(m), 852(m), 808(W), 742(w).

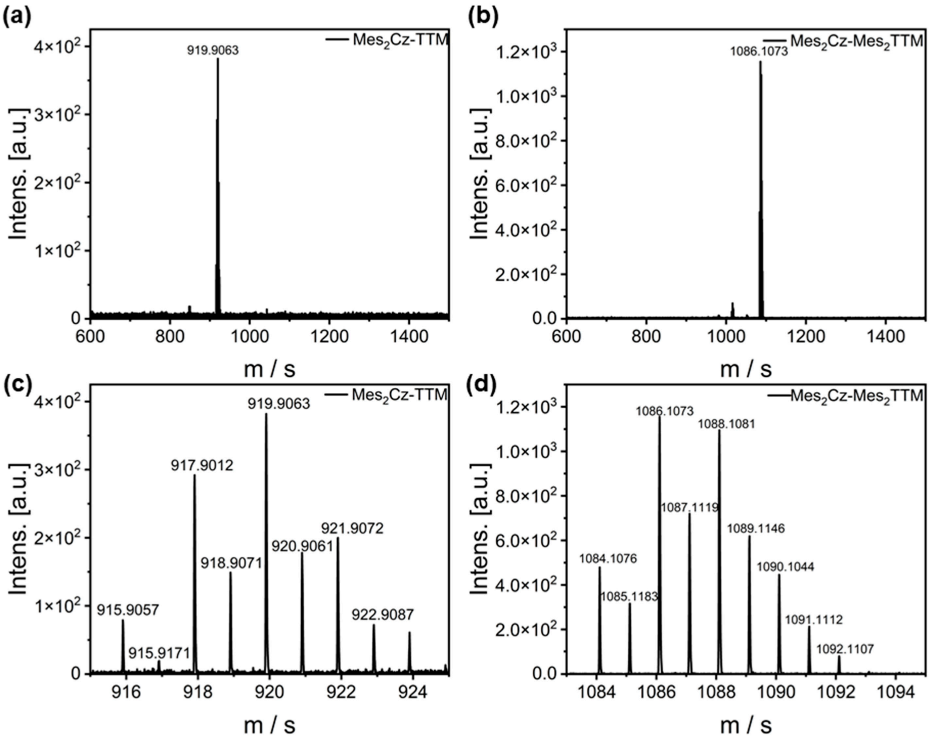

Figure S2. Mass spectrometry of Mes2Cz-TTM (a) (c) and Mes2Cz-Mes2TTM (b) (d).

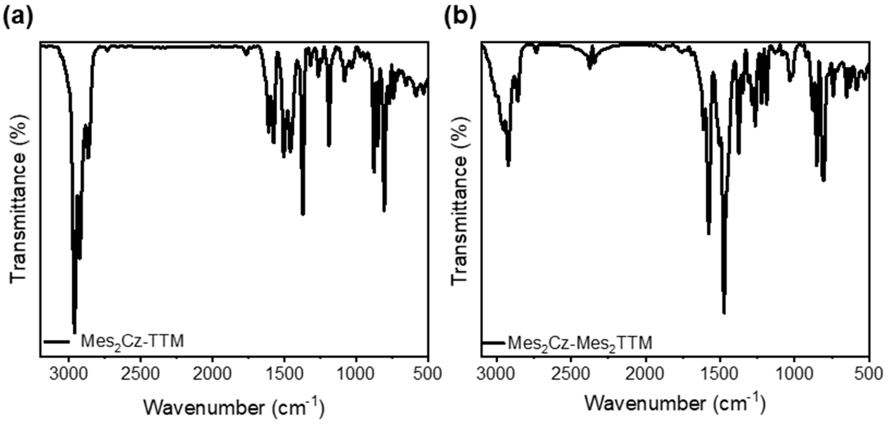

Figure S3. FT-IR spectra of Mes2Cz-TTM (a) and Mes2Cz-Mes2TTM (b).

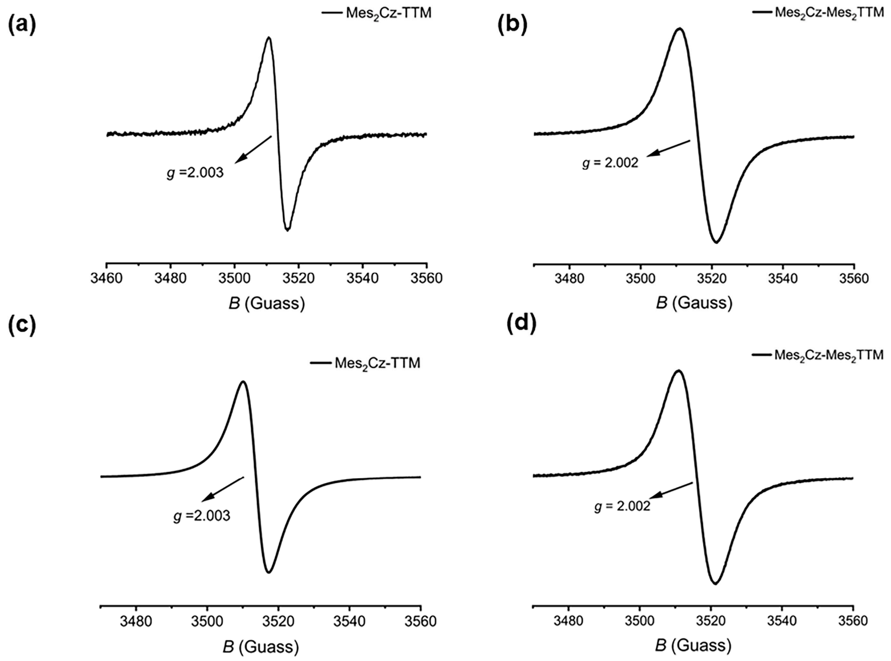

Figure S4. EPR spectra of radicals in dichloromethane solution form: Mes2Cz-TTM (a), Mes2Cz-Mes2TTM (b); and in powder form: Mes2Cz-TTM (c), Mes2Cz-Mes2TTM (d) at room temperature.

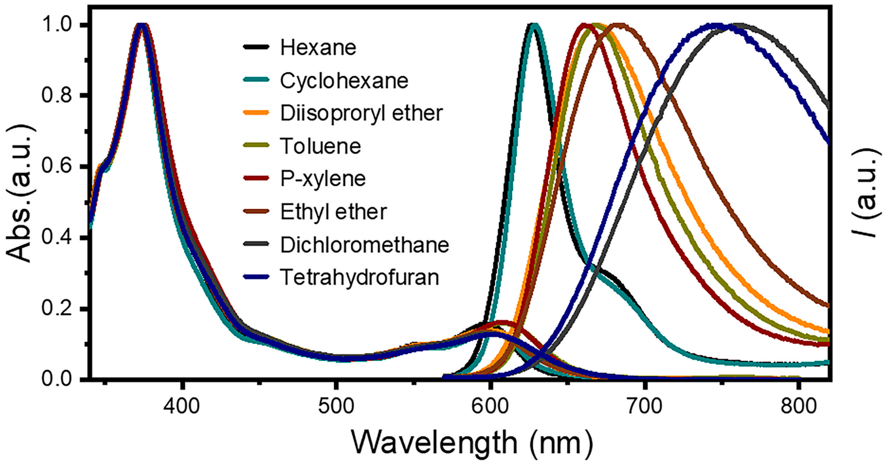

Figure S5. UV-Vis absorption spectra of Mes2Cz-TTM in solutions of different polarities (10^{-5} M).

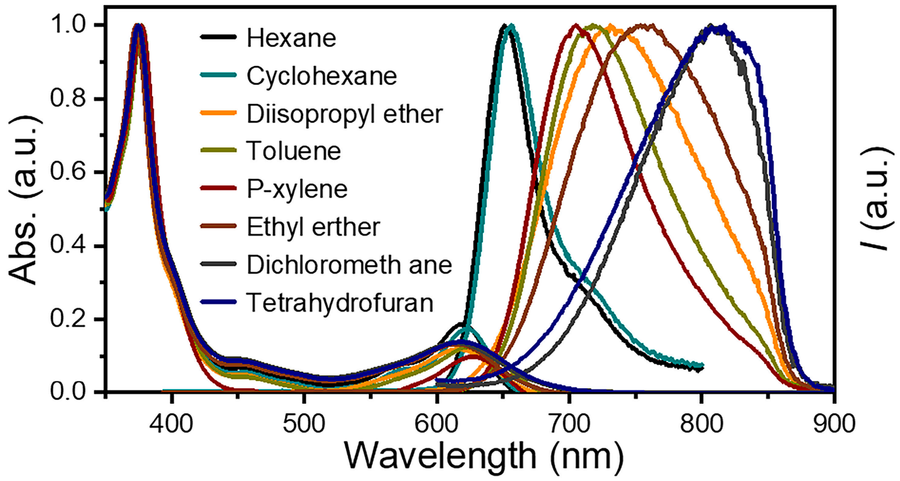

Figure S6. UV-Vis absorption spectra of Mes_2Cz-Mes_2TTM in solutions of different polarities (10^{-5} M).

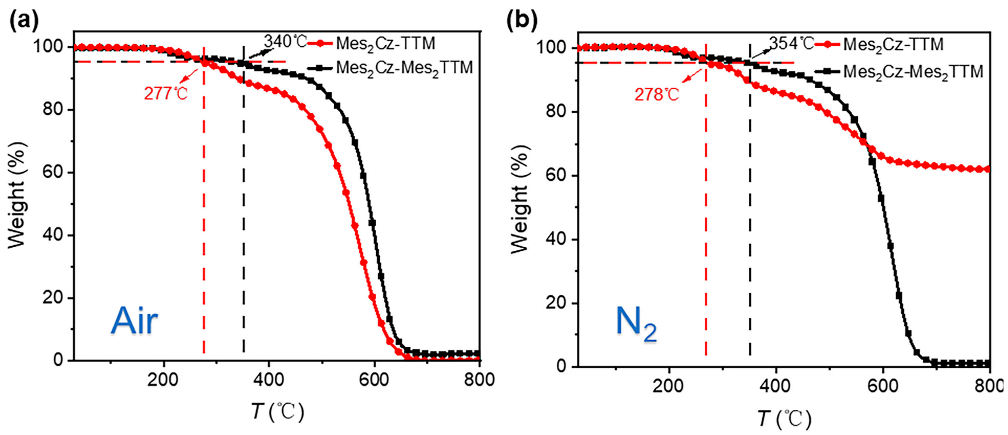

Figure S7. TGA curve of Mes_2Cz-TTM (a) and Mes_2Cz-Mes_2TTM (b).

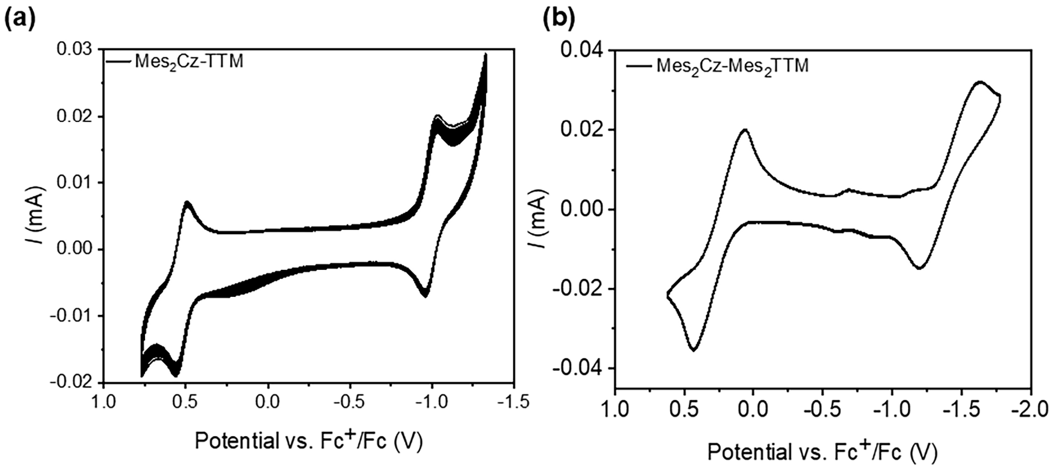

Figure S8 Cyclic Voltammetry (CV) curves of Mes_2Cz-TTM (a) and Mes_2Cz-Mes_2TTM (b) for multiple (20-turn) cycles.

Table S1. Redox potentials and corresponding orbital energy levels calculated theoretically and measured experimentally of Mes_2Cz-TTM and Mes_2Cz-Mes_2TTM.

Radicals	E_{ox}(V)	E_{red}(V)	α-SOMO		β-SUMO	
			Calculated	Experimental	Calculated	Experimental
Cz-TTM	0.55	-0.98	-5.45	-5.35	-3.36	-3.82
Mes_2Cz-TTM	0.53	-0.99	-5.42	-5.33	-3.36	-3.81
Mes_2Cz-Mes_2TTM	0.26	-1.40	-5.25	-5.06	-3.06	-3.40

Table S2. The values of characteristic torsion angles in radical molecules in theoretical calculations.

Radicals	Angle	Torsion angle（°）	
		GS [a]	OES[b]
Cz-TTM	α	47.8	41.2
	β	48.8	46.0
	γ	48.9	47.0
	θ	49.1	88.3
Mes_2Cz-TTM	α	47.7	41.6
	β	48.8	45.8
	γ	48.9	46.7
	θ	48.4	88.6
	δ	90.0	52.0
	φ	90.0	80.3
Mes_2Cz- Mes_2TTM	α	48.6	39.6
	β	48.8	46.4
	γ	48.8	48.0
	θ	50.8	88.1
	δ	90	52.9
	φ	89.6	98.8
	η	91.2	92.4
	ω	91.2	92.3

[a] Ground state; [b] Excited state; α, β and γ are the torsion angles of the two dichlorobenzene groups； θ are the torsion angles of the dichlorobenzene group and carbazole; δ and φ are the torsion angles of the benzene ring (2,4,6-trimethylbenzene) and carbazole； η and ω are the torsion angles of the the benzene ring (2,4,6-trimethylbenzene) and dichlorobenzene group.

Table S3. The bond lengths of radical molecules in theoretical calculations.

Radicals	Angle	Torsion angle（°）	
		GS [a]	OES[b]
Cz-TTM	C_1-C_2[c]	1.470	1.441
	C_1-C_8[c]	1.475	1.460
	C_1-C_{14}[c]	1.475	1.460
	C_1-N_1[d]	1.409	1.449
Mes_2Cz-TTM	C_1-C_2[c]	1.470	1.443
	C_1-C_8[c]	1.475	1.460
	C_1-C_{14}[c]	1.475	1.460
	C_1-N_1[d]	1.408	1.446
	C_{24}-C_{34}[e]	1.499	1.471
	C_{29}-C_{43}[f]	1.499	1.497
Mes_2Cz- Mes_2TTM	C_1-C_2[c]	1.473	1.434
	C_1-C_8[c]	1.475	1.464
	C_1-C_{14}[c]	1.475	1.464
	C_1-N_1[d]	1.411	1.446
	C_{24}-C_{34}[e]	1.499	1.497
	C_{29}-C_{43}[f]	1.499	1.497
	C_{11}-C_{52}[g]	1.497	1.497
	C_{17}-C_{61}[h]	1.497	1.493

[a] Ground state; [b] Excited state; [c] Bond length between the dichlorobenzene group and the central carbon atom; [d] Bond length between the dichlorobenzene group and carbazole; [e-f] Bond length between the the benzene ring (2,4,6-trimethylbenzene) and carbazole; [g-h] Bond length between the the benzene ring (2,4,6-trimethylbenzene) and dichlorobenzene group.

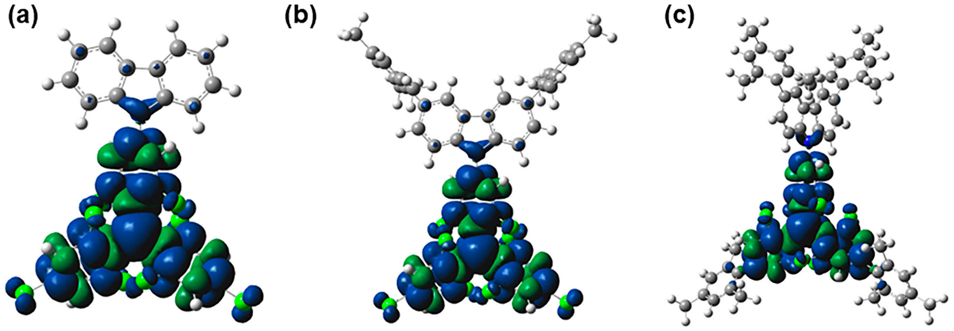

Figure S9. Spin densities of Cz-TTM (a), Mes_2Cz-TTM (b) and Mes_2Cz-Mes_2TTM (c) by DFT calculations.

Table S4. The parameters corresponding to the D_1 transition in the TD-DFT calculation results of radicals.

Radicals	Orbital excitation contribution	Transition energy (eV)	f
Cz-TTM	171β →172β （98%）	1.87	0.09
Mes_2Cz-TTM	235β →236β （98%）	1.83	0.11
Mes_2Cz-Mes_2TTM	283β →284β （98%）	1.99	0.1

Appendix (Calculation data)

Cz-TTM (UB3LYP/6-31G(d,p))

Center Number	Atomic Number	Atomic Type	Coordinates (Angstroms) X	Y	Z
1	6	0	-13.2458	-5.73133	0.128499
2	6	0	-10.6991	-6.4761	0.16804
3	6	0	-8.74891	-4.70674	0.119971
4	6	0	-9.41126	-2.1533	0.014362
5	6	0	-11.9704	-1.36839	0.011531
6	6	0	-13.8925	-3.183	0.061214
7	1	0	-14.7192	-7.158	0.16393
8	1	0	-10.2304	-8.4722	0.245067
9	1	0	-6.79119	-5.30613	0.18251
10	1	0	-15.8624	-2.60685	0.058708
11	6	0	-9.41112	2.154225	-0.01438
12	6	0	-8.74874	4.707644	-0.11963
13	6	0	-10.6989	6.477065	-0.1679
14	6	0	-13.2456	5.73239	-0.12886
15	6	0	-13.8924	3.184056	-0.06188
16	6	0	-11.9703	1.369435	-0.01202
17	1	0	-6.79103	5.307128	-0.1818
18	1	0	-10.23	8.473143	-0.24465
19	1	0	-14.719	7.159106	-0.16439
20	1	0	-15.8623	2.607903	-0.05979
21	7	0	-7.85585	0.000408	0.00017
22	6	0	-5.19383	0.000363	0.000104
23	6	0	-3.85166	-1.50194	-1.71122
24	6	0	-3.85166	1.502723	1.711412
25	6	0	-1.22879	-1.49712	-1.68538
26	1	0	-4.84866	-2.61118	-3.11096
27	6	0	-1.22881	1.497935	1.68553
28	1	0	-4.84871	2.612017	3.111064
29	6	0	0.228489	0.000317	0.000151
30	6	0	3.007711	0.000147	0.000234
31	6	0	4.404097	2.411617	0.025181
32	6	0	6.416078	2.905133	1.728427
33	6	0	3.845047	4.423705	-1.65814
34	6	0	7.755075	5.166693	1.763634
35	6	0	5.14484	6.708172	-1.65763
36	6	0	7.101154	7.059123	0.061683
37	1	0	9.25411	5.451671	3.125356
38	1	0	4.658279	8.16712	-3.00572
39	6	0	4.40333	-2.41192	-0.02467

40	6	0	3.843682	-4.42375	1.658656
41	6	0	6.41474	-2.90627	-1.72834
42	6	0	5.142374	-6.70887	1.657781
43	6	0	7.752562	-5.16851	-1.76402
44	6	0	7.098076	-7.0607	-0.06202
45	1	0	4.655412	-8.16762	3.005942
46	1	0	9.251154	-5.45418	-3.12608
47	17	0	0.22655	3.304829	4.057837
48	17	0	0.22659	-3.30331	-4.0582
49	17	0	1.541952	-4.09261	4.021759
50	17	0	7.251072	-0.7169	-4.07238
51	17	0	8.755967	-9.92097	-0.08472
52	17	0	1.542583	4.093598	-4.02065
53	17	0	7.251847	0.715547	4.072481
54	17	0	8.760523	9.918526	0.083722

Mes_2Cz-TTM (UB3LYP/6-31G(d,p))

Center Number	Atomic Number	Atomic Type	Coordinates (Angstroms) X	Y	Z
1	6	0	8.573623	5.751414	0.02986
2	6	0	6.005557	6.471594	0.055373
3	6	0	4.049135	4.709749	0.037112
4	6	0	4.69607	2.152521	-0.02474
5	6	0	7.253757	1.369815	-0.01297
6	6	0	9.179399	3.179899	0.00675
7	1	0	5.543967	8.470977	0.09748
8	1	0	2.095831	5.325006	0.08946
9	1	0	11.14845	2.59878	0.015222
10	6	0	4.696221	-2.15196	0.024168
11	6	0	4.04959	-4.70925	-0.0375
12	6	0	6.006217	-6.4709	-0.05564
13	6	0	8.574181	-5.75047	-0.03015
14	6	0	9.179685	-3.17887	-0.00723
15	6	0	7.253859	-1.36901	0.012347
16	1	0	2.096368	-5.32476	-0.08986
17	1	0	5.544853	-8.47034	-0.09762
18	1	0	11.14866	-2.59748	-0.01577
19	7	0	3.13816	0.000232	-0.00025
20	6	0	0.47723	0.00011	-0.00027
21	6	0	-0.86577	1.491323	-1.72112
22	6	0	-0.86555	-1.4912	1.72063
23	6	0	-3.48847	1.486375	-1.69521
24	1	0	0.130361	2.590565	-3.12922
25	6	0	-3.48828	-1.48646	1.694867

26	1	0	0.13072	-2.5903	3.128744
27	6	0	-4.94618	-0.00012	-9.1E-05
28	6	0	-7.72501	-0.00006	-6.6E-05
29	6	0	-9.12138	-2.41137	0.043545
30	6	0	-11.1327	-2.89216	1.751249
31	6	0	-8.56316	-4.43584	-1.62499
32	6	0	-12.4716	-5.15336	1.803851
33	6	0	-9.8629	-6.7203	-1.60699
34	6	0	-11.8184	-7.05843	0.115731
35	1	0	-13.9701	-5.42824	3.168296
36	1	0	-9.37684	-8.18924	-2.94437
37	6	0	-9.12101	2.411463	-0.0434
38	6	0	-8.56228	4.435832	1.625163
39	6	0	-11.1322	2.892755	-1.75101
40	6	0	-9.86134	6.720631	1.60707
41	6	0	-12.4706	5.154328	-1.80369
42	6	0	-11.8168	7.059291	-0.11569
43	1	0	-9.3749	8.189487	2.944403
44	1	0	-13.969	5.42952	-3.16811
45	17	0	-4.94293	-3.2768	4.08017
46	17	0	-4.94344	3.276617	-4.08037
47	17	0	-6.26104	4.12249	3.991326
48	17	0	-11.9674	0.685785	-4.07896
49	17	0	-13.4753	9.918898	-0.15991
50	17	0	-6.26168	-4.12321	-3.99099
51	17	0	-11.9671	-0.68508	4.079297
52	17	0	-13.4778	-9.91755	0.159869
53	6	0	10.61205	7.719291	0.044388
54	6	0	11.57693	8.628042	2.356817
55	6	0	11.56316	8.674984	-2.25449
56	6	0	13.47383	10.46834	2.327215
57	6	0	13.46039	10.51453	-2.19897
58	6	0	14.44792	11.43349	0.070715
59	1	0	14.20595	11.16656	4.117035
60	1	0	14.18181	11.24911	-3.97853
61	6	0	10.61198	-7.71901	-0.0445
62	6	0	11.56892	-8.66848	2.254638
63	6	0	11.58322	-8.62137	-2.35666
64	6	0	13.47422	-10.4995	2.199484
65	6	0	13.4883	-10.4533	-2.32671
66	6	0	14.45772	-11.423	-0.07024
67	1	0	14.20927	-11.2198	3.979346
68	1	0	14.23456	-11.1371	-4.11624
69	6	0	10.55929	7.748988	-4.76504

70	1	0	10.84029	5.711053	-4.99391
71	1	0	8.524513	8.084445	-4.94405
72	1	0	11.5042	8.703833	-6.33441
73	6	0	10.58879	7.650825	4.854213
74	1	0	8.554431	7.977921	5.051344
75	1	0	10.87511	5.609401	5.041648
76	1	0	11.54083	8.576634	6.436611
77	6	0	16.5403	13.37546	0.084783
78	1	0	18.40569	12.46967	0.091954
79	1	0	16.45675	14.59039	-1.58621
80	1	0	16.44108	14.58311	1.760352
81	6	0	10.60414	-7.63516	-4.85409
82	1	0	8.57012	-7.9602	-5.05795
83	1	0	10.89247	-5.59341	-5.03489
84	1	0	11.55989	-8.5573	-6.43639
85	6	0	10.57333	-7.73339	4.765116
86	1	0	10.85582	-5.6949	4.987193
87	1	0	8.538862	-8.0673	4.950419
88	1	0	11.52177	-8.68418	6.334806
89	6	0	16.47196	-13.4459	-0.0847
90	1	0	15.63233	-15.3419	-0.10984
91	1	0	17.68634	-13.293	-1.75127
92	1	0	17.67221	-13.3311	1.595084

Mes_2Cz- Mes_2TTM (UB3LYP/6-31G(d,p))

Center Number	Atomic Number	Atomic Type	Coordinates (Angstroms) X	Y	Z
1	6	0	-5.17633	0.000002	0.000013
2	6	0	-2.39234	0.000051	0.000026
3	6	0	-0.93599	-1.07608	1.97952
4	6	0	-0.93602	1.076225	-1.97947
5	6	0	1.688364	-1.10882	1.988062
6	6	0	1.688329	1.109037	-1.98802
7	6	0	3.027893	0.000132	0.000025
8	1	0	2.686412	-1.991	3.540432
9	1	0	2.686352	1.991235	-3.54039
10	6	0	-6.56989	2.353234	-0.5365
11	6	0	-6.01427	4.678954	0.67491
12	6	0	-8.58129	2.481766	-2.30379
13	6	0	-7.31995	6.904468	0.179016
14	6	0	-9.90942	4.691927	-2.80887
15	6	0	-9.2973	6.946735	-1.57381
16	1	0	-6.8111	8.609309	1.194052
17	1	0	-11.4106	4.659545	-4.20216

18	6	0	-6.56978	-2.3533	0.536522
19	6	0	-8.58118	-2.48192	2.303807
20	6	0	-6.01404	-4.679	-0.67488
21	6	0	-9.90919	-4.69216	2.808906
22	6	0	-7.31959	-6.90458	-0.17897
23	6	0	-9.29693	-6.94694	1.573868
24	1	0	-11.4104	-4.65985	4.202209
25	1	0	-6.81066	-8.6094	-1.19401
26	17	0	-3.70547	-4.85835	-3.05903
27	17	0	-9.42067	0.160627	4.134657
28	17	0	-3.70569	4.858433	3.059045
29	17	0	-9.42063	-0.16081	-4.13468
30	17	0	-2.39107	2.352845	-4.67538
31	17	0	-2.39099	-2.35272	4.675445
32	6	0	-10.7154	-9.33542	2.111578
33	6	0	-12.8573	-9.96465	0.656769
34	6	0	-9.91075	-10.9452	4.07653
35	6	0	-14.1566	-12.2016	1.19638
36	6	0	-11.2674	-13.1634	4.547949
37	6	0	-13.3913	-13.8311	3.128179
38	1	0	-15.8128	-12.6789	0.076283
39	1	0	-10.6459	-14.3993	6.068287
40	6	0	-10.7159	9.335128	-2.11147
41	6	0	-12.8571	9.964753	-0.65595
42	6	0	-9.91202	10.94444	-4.07715
43	6	0	-14.1566	12.20163	-1.19555
44	6	0	-11.2687	13.16259	-4.54851
45	6	0	-13.3921	13.83065	-3.12799
46	1	0	-15.8123	12.67924	-0.07489
47	1	0	-10.6478	14.39816	-6.06939
48	6	0	-7.62873	10.30707	-5.67318
49	1	0	-5.9241	10.09168	-4.51919
50	1	0	-7.8805	8.522107	-6.69117
51	1	0	-7.27063	11.78742	-7.06804
52	6	0	-13.7636	8.267051	1.456618
53	1	0	-14.2065	6.363876	0.774238
54	1	0	-12.3257	8.044691	2.929213
55	1	0	-15.4631	9.038197	2.341291
56	6	0	-14.79	16.26729	-3.63388
57	1	0	-14.0015	17.82787	-2.51929
58	1	0	-14.6647	16.81242	-5.6243
59	1	0	-16.7905	16.10931	-3.13663
60	6	0	-7.62681	-10.3083	5.671801
61	1	0	-5.92255	-10.0931	4.517252

62	1	0	-7.87796	-8.5233	6.689959
63	1	0	-7.26847	-11.7887	7.066461
64	6	0	-13.7645	-8.26643	-1.45506
65	1	0	-14.2077	-6.36358	-0.77195
66	1	0	-12.3269	-8.04325	-2.92782
67	1	0	-15.4639	-9.03763	-2.33968
68	6	0	-14.7892	-16.2677	3.633932
69	1	0	-16.7918	-16.1066	3.145984
70	1	0	-14.0073	-17.8261	2.51165
71	1	0	-14.6556	-16.8182	5.622321
72	6	0	7.25055	0.792417	1.998879
73	6	0	7.250516	-0.79216	-1.99888
74	6	0	6.600188	1.810943	4.344501
75	6	0	9.809801	0.507082	1.27189
76	6	0	6.600114	-1.81067	-4.3445
77	6	0	9.809778	-0.50685	-1.27193
78	6	0	8.554276	2.493815	5.970954
79	1	0	4.644947	2.084997	4.891811
80	6	0	11.73313	1.209582	2.943587
81	6	0	8.554176	-2.49356	-5.97098
82	1	0	4.644864	-2.0847	-4.89179
83	6	0	11.73308	-1.20937	-2.94365
84	6	0	11.12367	2.201317	5.31514
85	1	0	8.09066	3.285304	7.807073
86	1	0	13.70313	0.998274	2.405166
87	6	0	11.12358	-2.20109	-5.3152
88	1	0	8.090528	-3.28504	-7.80709
89	1	0	13.70309	-0.99809	-2.40524
90	7	0	5.695166	0.000147	0.000004
91	6	0	13.1583	-2.96006	-7.13522
92	6	0	14.12021	-5.44553	-7.10563
93	6	0	14.12177	-1.18891	-8.87832
94	6	0	16.02175	-6.1125	-8.81622
95	6	0	16.02325	-1.93402	-10.5562
96	6	0	16.99701	-4.38827	-10.5618
97	1	0	16.76041	-8.03088	-8.77944
98	1	0	16.7629	-0.55699	-11.8919
99	6	0	13.15842	2.960273	7.13513
100	6	0	14.12196	1.189106	8.87816
101	6	0	14.12031	5.445756	7.105563
102	6	0	16.02349	1.934193	10.55604
103	6	0	16.0219	6.11271	8.816101
104	6	0	16.99722	4.388451	10.56166
105	1	0	16.76319	0.557137	11.89162

106	1	0	16.76055	8.031094	8.779339
107	6	0	13.13462	-7.3866	-5.25392
108	1	0	13.42322	-6.787	-3.29394
109	1	0	11.0998	-7.69139	-5.47957
110	1	0	14.08538	-9.20228	-5.51329
111	6	0	13.13606	1.492655	-8.9528
112	1	0	11.10165	1.546168	-9.33119
113	1	0	13.42195	2.461542	-7.14619
114	1	0	14.08829	2.587889	-10.423
115	6	0	19.00693	-5.16807	-12.4332
116	1	0	20.21107	-6.66613	-11.6706
117	1	0	18.16355	-5.89122	-14.1843
118	1	0	20.21828	-3.57426	-12.9511
119	6	0	13.13627	-1.49247	8.95263
120	1	0	11.10187	-1.54601	9.331039
121	1	0	13.42215	-2.46134	7.146007
122	1	0	14.08854	-2.58772	10.42283
123	6	0	13.13464	7.38686	5.253932
124	1	0	13.42313	6.78728	3.293929
125	1	0	11.09983	7.691652	5.479686
126	1	0	14.08542	9.202522	5.513272
127	6	0	19.00722	5.168225	12.43298
128	1	0	18.16393	5.891559	14.18401
129	1	0	20.21846	3.574366	12.95097
130	1	0	20.21147	6.666147	11.67026

Supporting Reference

[1] R. Xiaotian, W. Ota, T. Sato, M. Furukori, Y. Nakayama, T. Hosokai, E. Hisamura, K. Nakamura, K. Matsuda, K. Nakao, A. P. Monkman, K. Albrecht, Angew. Chem. Int. Ed. **2023**, 62, e202302550.
